# Habitat traits and predation interact to drive abundance and body size patterns in associated fauna

**DOI:** 10.1002/ece3.10771

**Published:** 2023-12-03

**Authors:** Talia P. Stelling‐Wood, Alistair G. B. Poore, A. Randall Hughes, Jason D. Everett, Paul E. Gribben

**Affiliations:** ^1^ Evolution & Ecology Research Centre UNSW Sydney Sydney New South Wales Australia; ^2^ Centre of Marine Science and Innovation UNSW Sydney Sydney New South Wales Australia; ^3^ Marine Science Center Northeastern University Nahant Massachusetts USA; ^4^ School of Mathematics and Physics The University of Queensland St Lucia Queensland Australia; ^5^ Sydney Institute of Marine Science Mosman New South Wales Australia

**Keywords:** habitat structure, intraspecific variation, macroalgae, *Sargassum vestitum*, trait‐based ecology

## Abstract

Habitat‐forming organisms provide three‐dimensional structure that supports abundant and diverse communities. Variation in the morphological traits of habitat formers will therefore likely influence how they facilitate associated communities, either via food and habitat provisioning, or by altering predator–prey interactions. These mechanisms, however, are typically studied in isolation, and thus, we know little of how they interact to affect associated communities. In response to this, we used naturally occurring morphological variability in the alga *Sargassum vestitum* to create habitat units of distinct morphotypes to test whether variation in the morphological traits (frond size and thallus size) of *S. vestitum* or the interaction between these traits affects their value as habitat for associated communities in the presence and absence of predation. We found morphological traits did not interact, instead having independent effects on epifauna that were negligible in the absence of predation. However, when predators were present, habitat units with large fronds were found to host significantly lower epifaunal abundances than other morphotypes, suggesting that large frond alga provided low‐value refuge from predators. The presence of predators also influenced the size structure of epifaunal communities from habitat units of differing frond size, suggesting that the refuge value of *S. vestitum* was also related to epifauna body size. This suggests that habitat formers may chiefly structure associated communities by mediating size‐selective predation, and not through habitat provisioning. Furthermore, these results also highlight that habitat traits cannot be considered in isolation, for their interaction with biotic processes can have significant implications for associated communities.

## Graphical abstract

We used naturally occurring morphological variability in the alga *Sargassum vestitum* to create habitat units of specific morphotypes and found that intraspecific variation in frond size strongly affected the epifaunal communities associated with macroalgae. Experimentally excluding predators showed that these patterns were likely the result of predation, with epifaunal communities only varying in the presence of predators. This suggests that algal morphology affects the refuge value of macroalgae for associated epifauna.

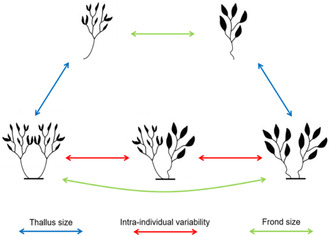

## 1 INTRODUCTION

Interactions amongst organisms are one of the most important drivers of the distribution and abundances of species around the world (Jones et al., 1994). Habitat‐forming organisms (hereafter habitat formers) are of particular importance for such interactions as they can regulate the availability of resources to other organisms (Teagle et al., 2017). They structure communities by creating, modifying and maintaining habitat, as well as altering biotic interactions (e.g. predation) and abiotic stressors (e.g. thermal and wave stress) (Bertness et al., 2001; Klecka & Boukal, 2014; Romero et al., 2015; Wright & Gribben, 2017). Consequently, widespread habitat formers such as trees, corals and macroalgae support highly abundant and diverse communities (Bruno et al., 2005; Lapointe & Bourget, 1999; MacArthur & MacArthur, 1961).

Recent research has confirmed long‐standing predictions about the pervasive role of habitat structure in shaping the communities that are associated with habitat formers (Thomsen et al., 2022). Generally, more complex or heterogeneous habitats (e.g. a high number and/or diversity of structural elements) are associated with higher abundances and more diverse communities, due to the increased number and/or greater diversity of niches they provide (Khanaposhtani et al., 2012; MacArthur & MacArthur, 1961). Habitat complexity can also, however, affect the ability of predators to detect and capture prey (Denno et al., 2005). Consequently, the structure of communities associated with habitat formers is a function of both abiotic provisioning of habitat structure (sensu Jones et al., 1994) and altered biotic interactions. For example, mollusc shells enhance species richness and abundance by providing necessary structure for the settlement of sessile invertebrates, but this community‐level facilitation can be reduced in the presence of predators (Gribben et al., 2017). Commonly, however, studies only report the net effects of changes in habitat structure on associated communities, and thus, we know little about the relative contribution of habitat provisioning and biotic processes, or their possible interaction on these communities.

The relative importance of habitat structure and predation for associated communities is also likely to vary with the body size of organisms relative to the structural features of the habitat former (Gee & Warwick, 1994). For example, a mismatch between an organism's body size and the size of a microhabitat will influence the value of the habitat former as living space (McAbendroth et al., 2005). When predators are present, however, the value of habitat formers will also depend on the ability of microhabitats to host prey individuals while simultaneously excluding predators (Bartholomew et al., 2000; Toscano & Griffen, 2013). Different‐sized organisms will therefore utilise habitat differently (Raffaelli et al., 2000), and consequently, habitat structure can strongly affect the size structure of benthic communities (Robson et al., 2005; Schwinghamer, 1981). Peaks and troughs in abundance (or biomass) along a body size axis are driven by the uneven distribution of resources within a habitat, as organisms will aggregate where resources are freely available (peaks) and will be separated by gaps (troughs) where resources are limited (Holling, 1992). Thus, considerations of the relationship between body size and abundance can also be used to inform questions about secondary productivity and trophic dynamics, and how these might vary in relation to habitat structure (Heather et al., 2021).

On temperate reefs, macroalgae are the dominant habitat formers, providing three‐dimensional structures that support abundant and diverse communities (Bertness et al., 2001; Lloyd et al., 2020). Temperate reefs also, however, support large fish populations which prey upon invertebrate communities (Edgar & Aoki, 1993). A recent study including multiple, morphologically diverse algae found that the trait frond size ranked as more important than species identity when predicting the density of macroalgal associated epifauna (Stelling‐Wood et al., 2020). We therefore selected one species of macroalgae that displays high phenotypic plasticity in frond size, *Sargassum vestitum* (R. Brown ex Turner) C. Agardh, to test hypotheses about this structural trait independent of species identity. Traits can, however, also interact, with their effects being either independent (i.e. no effect or additive effect) or dependent on each other (i.e. synergistic or antagonistic effect). In the case of habitat formers, morphological trait interactions could influence their value to the associated organisms as habitat. Despite this, multiple traits are rarely considered in trait‐based approaches, with the possible interaction between multiple traits considered even less frequently (Phillips & Arnold, 1989) despite often leading to more realistic models with increased predictive accuracy (Laughlin, 2014; Pistón et al., 2019). We therefore tested whether variation in the morphological traits of *S. vestitum*, or the interaction between traits, affect their value as habitat for associated communities by creating habitat units that varied in two morphological traits, frond size and thallus size, testing for differences in the number of invertebrates that colonised habitat units.

The structure of macroalgae is important not only as habitat but also in mediating predator–prey interactions on temperate reefs (Zamzow et al., 2010) meaning that the structural traits of macroalgae can influence their value as refuge if predators are present. Cages were used to manipulate predator access to macroalgal associated epifaunal communities to test the prediction that predation risk and algal morphology interact, with increased structural complexity reducing the risk of associated organisms to predation. This design permitted us to separate out the relative influence of abiotic habitat provisioning and mediation of the biotic interaction predation risk in structuring macroalgal associated epifaunal communities. To assess associated community responses, we quantified total abundance, mean body length, variance in body length and the size structure of the epifaunal communities associated with *S. vestitum*. We predicted the abundance of organisms associated with macroalgae will increase with thallus size, and that both abundance and body size diversity will increase with morphological trait diversity. Furthermore, we predicted that variation in morphological traits will have a stronger effect on associated communities in the presence of predators.

## 2 METHODS

### 2.1 Study area and field collection

The genus *Sargassum* is known to display high naturally occurring morphological variability (Coleman & Wernberg, 2017; Stelling‐Wood et al., 2020). *S. vestitum* is a foliose, perennial brown alga that dominates temperate coastlines around Australia. *S. vestitum* is known to display high morphological variation, both between individuals (intraspecific variation) but also within individual thalli (intra‐individual variation) (Stelling‐Wood et al., 2020, 2021). This permitted us to investigate the relationship between habitat structure and associated fauna without being confounded by species‐specific traits.

**FIGURE 1:**
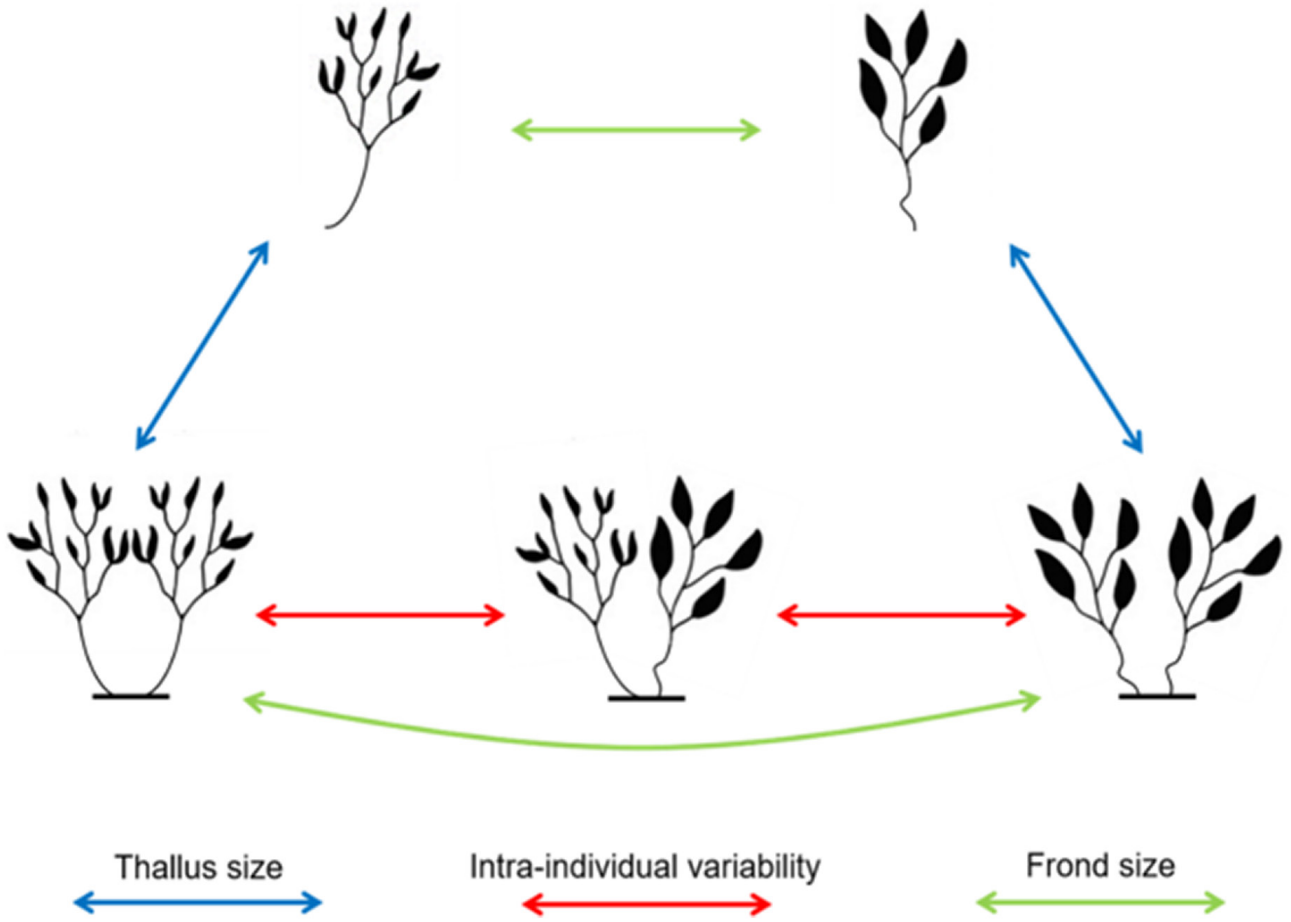
The contrasts among treatments used to test for interactive trait effects on epifauna. The five habitat treatments were (1) single branch small frond habitat unit, (2) single branch large frond habitat unit, (3) double branch small frond habitat unit, (4) double branch large frond habitat unit and (5) double branch mixed frond habitat unit. Comparisons between the single branch treatments tested for the effect of frond size trait alone on associated communities (green arrow). Comparisons among double branch habitat units of single morphotype (small and large, but not mixed) also test for the effect of frond size trait alone (green arrow). Comparison of single branch habitat units and double branch habitat unit of single morphotype (between small and large only) tested for the effect of frond size combined with thallus biomass (blue arrows). Comparisons among all morphotypes of double branch habitat units (small, large and mixed) tested for the effect of intra‐individual morphological variability on associated communities (red arrows).

**FIGURE A1:**
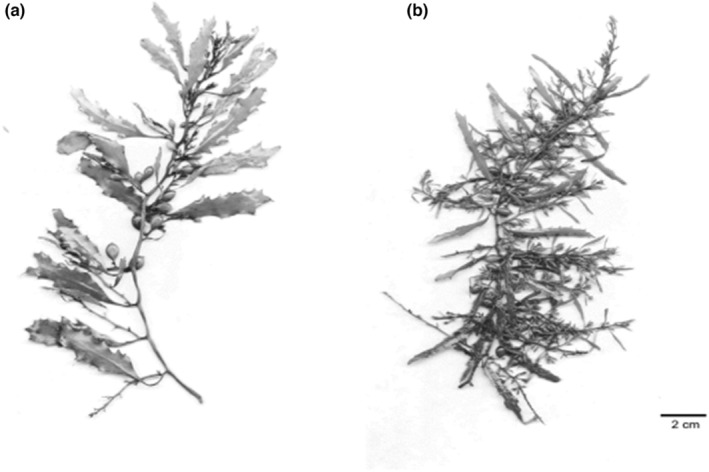
Representative image of the Sargassum vestitum branches used in this experiment, (a) large frond morphotype and (b) small frond morphotype.

Algal material (*S. vestitum* thalli) for habitat units was collected on snorkel from Shark Bay in Sydney Harbour, New South Wales, Australia (33°51′ S, 151°14′ E) in November 2018. Eight large thalli were collected and once back on shore were placed into freshwater for ~10 min to remove all resident epifauna (Machado et al., 2019). Branches were then separated from the holdfast and the top 15 cm sections were removed. Each branch was visually separated into ‘small’ (frond length < 35 mm) and ‘large’ (frond length > 40 mm) frond morphotypes (Figure A1). The visual allocation of morphotypes was validated upon retrieval of the experiment (see Appendix A for details). We then created and deployed five types of habitat units: (1) a single branch with small fronds, (2) a single branch with large fronds, (3) two branches with small fronds, (4) two branches with large fronds and (5) a unit with one branch with small fronds and one branch with large fronds (hereafter ‘mixed frond’ in text and ‘mix’ in plots) (Figure 1). Habitat units with a single branch consisted of one 15 cm branch of algae (hereafter single branch habitat unit), whereas habitat units with two branches had two 15 cm branches connected with a cable‐tie at the base (hereafter double branch habitat unit).The high intra‐individual morphological variability displayed by *S. vestitum* meant that each individual thalli collected could potentially contribute algal material to any habitat unit (small, large and mixed mean frond size, and both single‐ and double‐branched habitat units) and were therefore randomly allocated among all habitat unit morphology treatments. All habitat units was then kept in saltwater until deployment later that day.

To test whether habitat unit size (single‐ or double‐branched habitat units) interacted with frond size diversity (small, large and mixed mean frond size) to affect the abundance and size structure of associated communities all five habitat unit morphotypes were deployed simultaneously and colonising epifaunal communities were compared (*n* = five to six habitat units for each of the five morphotypes).

To test how variation in algal morphology and predation risk structure epifaunal communities, we used an orthogonal design with predation risk (three levels) and habitat unit morphology (three levels, as only double‐branched habitat units were used); with six replicates for each combination level. The predation risk treatments employed full cages (excludes predators >1 cm wide), open cages (allows predator access, but controls for possible cage effects) and uncaged (allows predator access). Cylindrical cages (18 cm high × 15 cm in diameter) were constructed of plastic gutter guard with 1 cm^2^ mesh size. The open cages had two openings (10 cm × 5 cm), on opposite sides of the cage.

Habitat units were randomly allocated among treatments. Each treatment, consisting of one habitat unit plus one cage (full‐ and open‐cage treatments only), was then attached with cable ties to one corner of a plastic mesh ‘patch’ (four treatments per patch). A patch consisted of a 50 cm × 50 cm square of plastic mesh with one concrete masonry brick (390 × 190 × 190 mm, ~13.3 kg) attached to the centre of the mesh patch with cable ties. To account for possible variation among patches, treatments were randomly assigned among different patches so that it was possible for any combination of treatments to occur on each patch. *S. vestitum* has naturally occurring vesicles (air sacks) to keep branches neutrally buoyant and upright therefore there was no need to attach floats to habitat units. Each patch of mesh was then floated out to the algal bed on a floatation device and was gently lowered to the seafloor. Patch locations were haphazardly selected; however, only flat surfaces were used to ensure algae remained upright and a minimum of 1 m between patches was maintained to ensure independence (as per Roberts & Poore, 2006). All naturally occurring algae around patches were removed to ensure no macrophytes were touching the habitat units (all treatments were a minimum of 50 cm to the nearest algal bed).

After 5 days, all algae were retrieved by snorkelling—a time period that allows colonisation of mobile epifauna to close to natural densities at this study site (Poore, 2005; Roberts & Poore, 2006). Each branch was collected separately by removing cage (if present); then the cable tie at the attachment point and quickly placing each branch of the habitat unit in a jar underwater (i.e. for double branch habitat units branches were placed in separate jars). Back in the laboratory, all jars were stored in the refrigerator (~2°C) until processing (maximum 3 days). To quantify the mobile epifaunal community on each sample, the algae were rinsed three times with tap water, then poured over a 200‐μm sieve to capture epifauna. The epifauna sample was then scanned using ZooScan (ZooScan MIII, Hydroptic Inc., France), an industrialised, water‐resistant high‐resolution scanner for organisms ranging in size from 200 μm to several centimetres (Gorsky et al., 2010). To prepare samples, each sample was suspended in tap water and then poured into the ZooScan. Images were then scanned at 1200 dpi. Once scanned, images were processed in ImageJ (1.50i; National Institute of Health, Bethesda, MD) to count and measure (total body length: μm) all epifauna.

A generalised linear model (GLM) was used to test for differences in the abundance of epifauna that colonised habitat units across all five morphology treatments. In these models, algal biomass was included as an offset to account for differences in the biomasses of habitat units. A Poisson error distribution was used. Similar analyses were run with mean body size and variance in body size as response variables. Mean body size was, however, analysed using a linear model, while variance in body size was analysed using a GLM with a Gamma log link error distribution. Another GLM which used a factorial design was used to test for an interaction between frond size and habitat unit size on the abundance of epifauna by excluding the mixed frond double‐branched habitat units. For all these analyses, one entire habitat unit was the smallest unit of measurement (i.e. both branches were included for double‐branched habitat units).

**FIGURE 2:**
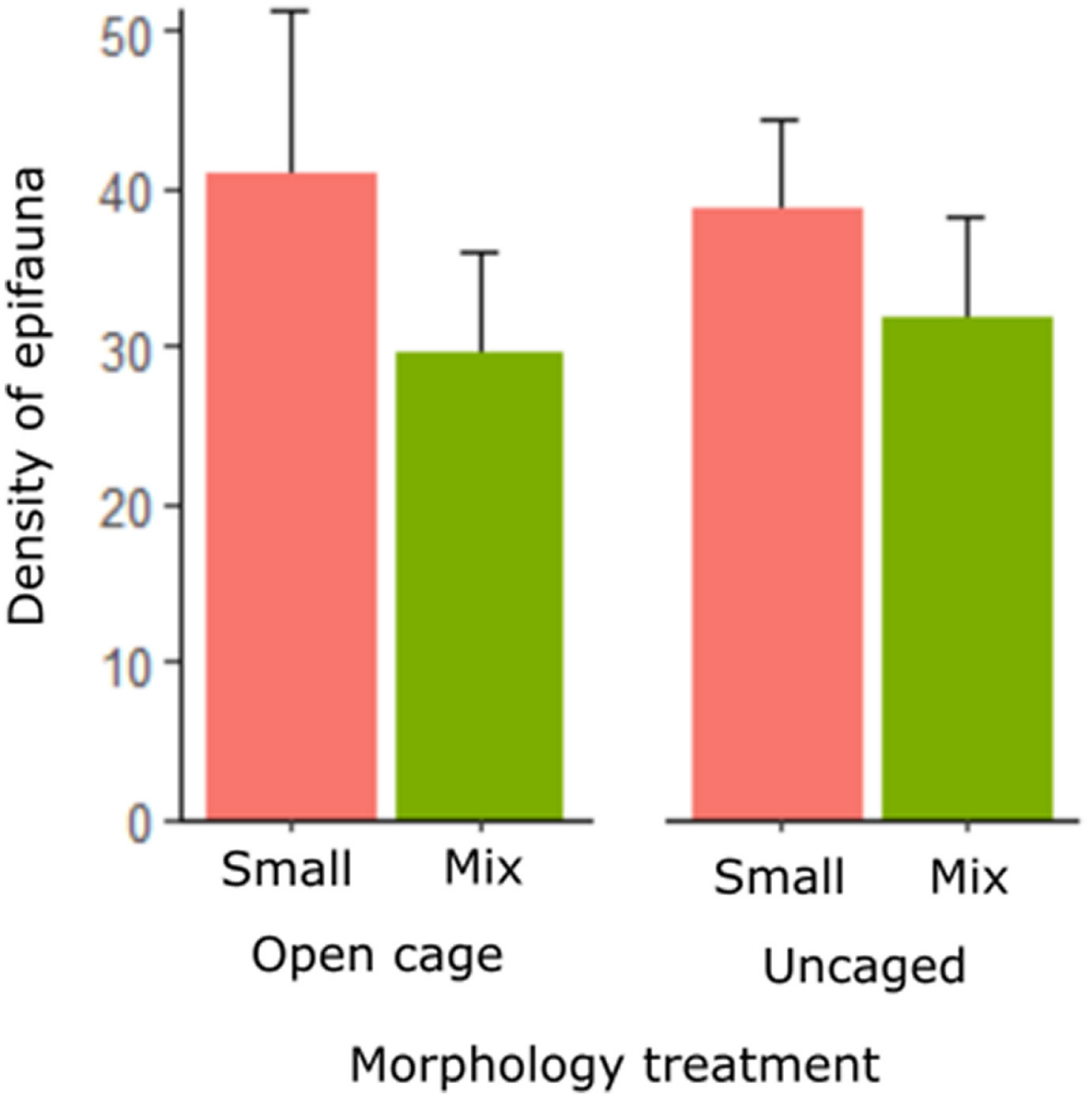
Caging artefact analysis test for the effect of the presence of caging material on epifaunal communities. ‘Small’, small frond double stand habitat units and ‘Mix’, mixed frond double branch habitat units. ‘Density of epifauna’ is abundance per gram biomass of algal material. Large frond habitat units could not be included due to the loss of several large frond habitat units to suspected herbivory. This analysis therefore compares open‐cage treatments to uncaged treatments for small and mixed frond habitat units only. Analyses showed no significant differences between open‐cage and uncaged treatments for small frond habitat units (*p* = .59, Table 1) and only slight differences between mixed frond habitat units for the abundance of epifauna (*p* = .01, Table 1). Error bars are standard error. Different letters indicate significant differences in the abundance of epifauna in models using algal biomass as an offset.

Suspected herbivory during these experiments resulted in only one replicate of the large frond habitat units remaining in open‐cage treatments. Analyses showed no significant differences between open‐cage and uncaged treatment for small frond double branch habitat units and only slight differences between mixed frond double branch habitat units for the abundance of epifauna (*p* = .01, Table 1, Figure 2). Therefore, to maintain an orthogonal design that would allow us to test for the interaction between habitat unit morphology and predation risk, open‐cage treatments were excluded from further analyses. We used a GLM to test for an interaction between habitat unit morphology and cage treatment (full‐cage and uncaged treatments only) on the abundance of epifauna using a Poisson error distribution. Significant effects of treatments on mean body length were also tested using LMs, while the effect of treatments on the variance in body length was tested using GLMs with a Gamma log link error distribution.

**TABLE 1:** Caging artefact analysis test for the effect of the presence of caging material on epifaunal communities.

	Abundance			Mean body length			Variance in body length		
	Df	Z	*P*(<Z)	Df	Z	*P*(<Z)	Df	Z	*P*(<Z)
Small	1,10	.29	.59	1,10	4.13	.14	1,10	.35	.11
Mix	1,9	6.11	.01*	1,9	.22	.77	1,9	.06	.71

*Note*: ‘Small’ = small frond double branch habitat units and ‘Mix’ = mixed frond double branch habitat units. Open‐cage treatments could not be included due to the loss of several large frond habitat units to suspected herbivory. This analysis therefore compares open‐cage treatments to uncaged treatments only. Algal biomass was included as an offset in the model testing for an effect on epifauna abundance. Abundance was tested using a GLM with a Poisson error distribution, while mean body length was tested using an LM and variance in body length was tested using a GLM with a Gamma error distribution with a log link function. Significant differences are indicated with *. See Figure 2 for caging artefact plot for the abundance of epifauna.

The size structure of epifaunal communities was visualised using frequency histograms on log‐transformed body length measurements. Delta plots (Wang et al., 2022) were used to explore differences in the distribution of epifaunal community body sizes (size structure) across the different treatment combinations. Replicates were pooled across treatments and the uncaged treatment was used as the baseline to represent ‘natural’ community size structure. All lines on the delta plots, therefore, represent differences in the size structure of epifaunal communities in a given treatment compared to that baseline (i.e. communities from uncaged treatments).

All data visualisation and statistics were done in R v3.6.3 (R Core Team, 2020). Models were visually assessed for heteroscedasticity (Zuur et al., 2009). Tukey's post hoc test in the r package ‘emmeans’ was used to assess differences amongst groups of significant factors (Lenth et al., 2018).

## 3 RESULTS

**FIGURE 3:**
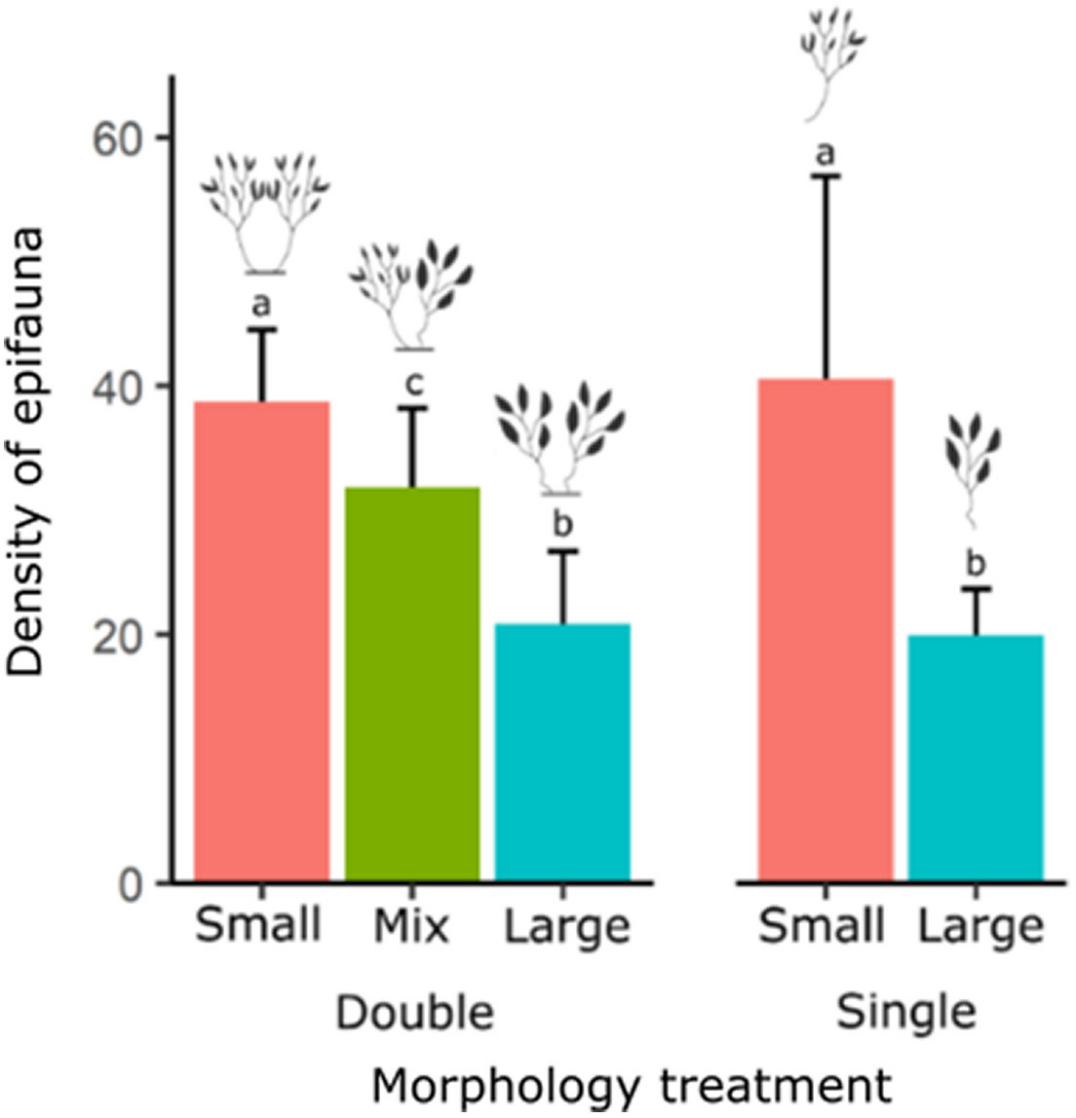
Effect of habitat unit morphology on the density of epifauna. Different colours reflect frond size diversity of habitat units (small, large and mixed mean frond size), while ‘double’ and ‘single’ represent habitat unit size (single or double branch habitat units). ‘Density of epifauna’ is abundance per gram biomass of algal material. Error bars are standard error. Different letters indicate significant differences in the abundance of epifauna in models using algal biomass as an offset.

A total of 15,019 individuals was found associated with our habitat units. Epifauna colonised all treatments after 5 days to densities that approximated or exceeded natural densities (natural densities taken from Stelling‐Wood et al., 2020). Analyses showed that the abundance of epifauna varied significantly with habitat unit morphology (Figure 3 & Table 2a). Pairwise comparisons among treatments showed that small frond habitat units (single‐ and double‐branched) had higher densities than mixed frond double‐branched habitat units (*p* < .0001), which had higher densities than large frond habitat units (single‐ and double‐branched, *p* < .0001), suggesting that there was no interactive effect of frond size traits on the epifauna abundance. There were no significant differences between single‐ and double‐branched habitat units of the same frond size (small frond habitat units: *p* = .35 & large frond habitat units: *p* = .55). The results from the orthogonal analyses supported this, with no significant interaction between frond size and habitat unit size (Table 3). There was no significant effect of habitat unit morphology on the mean body size or variability in body size of epifaunal communities (Table 4).

**TABLE 2:** Results of models testing for (a) the influence of habitat unit morphology and (b) the effects of habitat unit morphology and predator access (cage treatment) on the abundance of epifauna per habitat unit.

	Df	Z	*P*(<Z)
(a) Habitat unit morphology	4,24	−355.8	<.0001*
(b) Cage treatment (C)	1,30	38.3	<.0001*
Habitat unit morphology (HM)	2,30	149	<.0001*
C × HM	2,27	130.8	<.0001*

*Note*: Open‐cage treatments could not be included in an orthogonal design due to the loss of several large frond habitat units to suspected herbivory. This analysis, therefore, compares open‐cage treatments to uncaged treatments only. Algal biomass was included as an offset and a Poisson error distribution was used. Significant differences are indicated with *.

**TABLE 3:** Results of model testing for an interaction between frond size and habitat unit size on the abundance of epifauna per habitat unit, by excluding the mixed double branch treatment.

	Df	Z	*P*(<Z)
Frond size (FS)	1,19	−349.61	.0001*
Habitat unit size (HS)	1,19	−7.05	.01*
FS × HS	1,17	−.002	.96

*Note*: Algal biomass was included as an offset. Significant differences are indicated with *.

**TABLE 4:** Results of model testing for the effect of habitat unit morphology on mean body length and variance in body length of epifauna communities.

	Mean body length			Variance in body length		
	Df	Z	*P*(<Z)	Df	Z	*P*(<Z)
Habitat unit morphology	4,24	−3.88	.57	4,24	−1.9	.26

*Note*: Mean body length was tested using an LM and variance in body length was tested using a GLM with a Gamma error distribution with a log link function.

**FIGURE 4:**
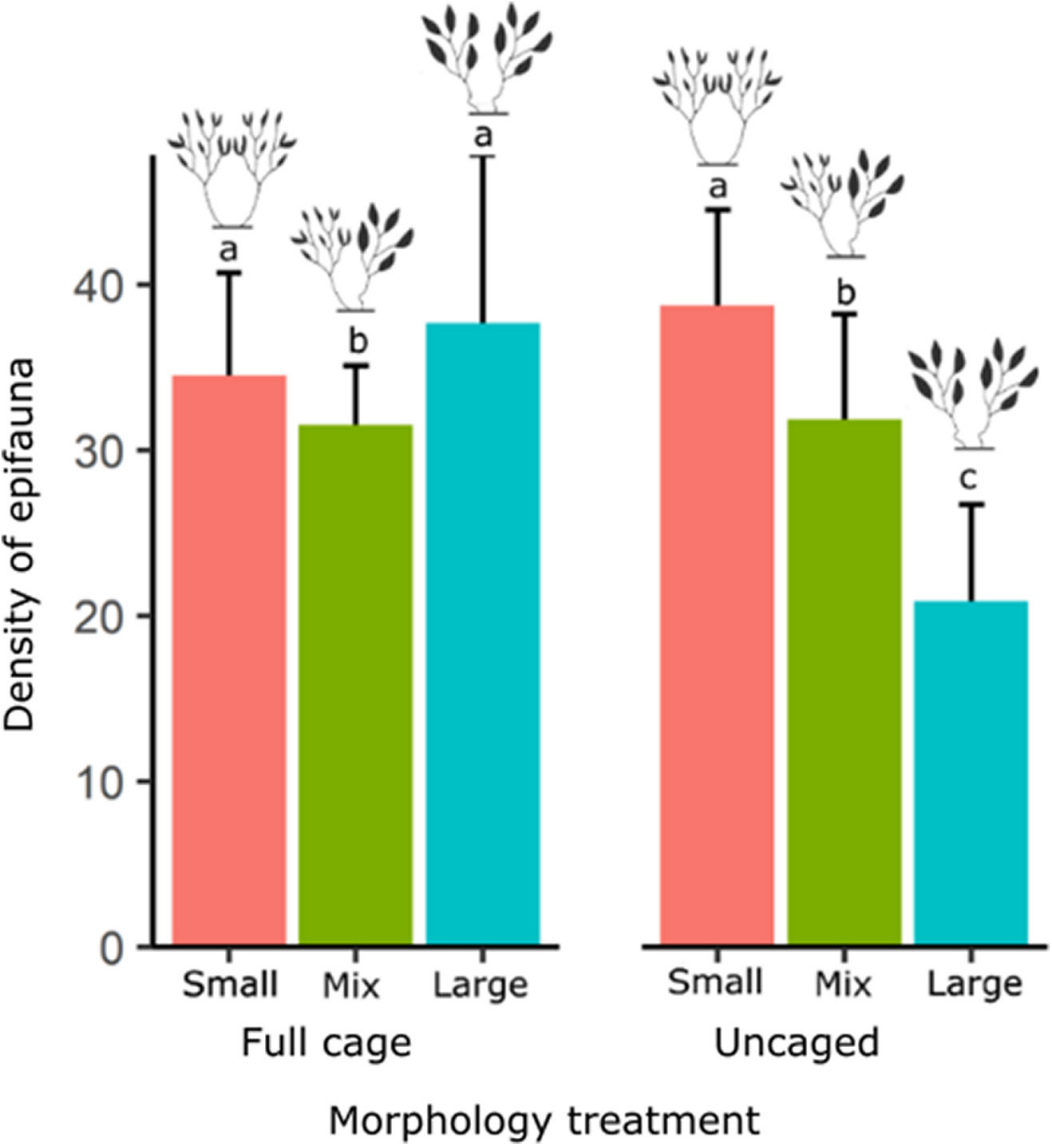
Effect of habitat unit morphology and predator access (cage treatment) on the density of epifauna. Different colours reflect frond size diversity of habitat units (small, large & mixed frond size). ‘Density of epifauna’ is abundance per gram biomass of algal material. Open‐cage treatments could not be included in an orthogonal design due to the loss of several large frond habitat units to suspected herbivory (see main text for more details). This analysis therefore compares open‐cage treatments to uncaged treatments only. Error bars are standard error. Different letters indicate significant differences in the abundance of epifauna in models using algal biomass as an offset.

Comparing full‐cage and uncaged treatments only, there was a significant interaction between caging treatment and habitat unit morphology (Table 2b). Within full‐cage treatments, mixed frond habitat units had slightly but significantly lower densities than both large (*p* = .0002) and small (*p* = .001) frond habitat units (Figure 4, Table 5). The effect of morphology, however, was much stronger in uncaged treatments (Figure 4). While small and mixed frond habitat units from uncaged treatments did not differ from habitat units of the same morphology in full‐cage treatments (small frond habitat units: uncaged × full cage, *p* = .21; mixed frond habitat units: uncaged × full cage, *p* = .88, Table 5), large frond habitat units in uncaged treatments had significantly lower abundances of epifauna than large frond habitat units in full‐cage treatments (*p* < .0001) (Figure 4, Table 5). Similarly, large frond habitat units in uncaged treatments also hosted significantly lower abundances of epifauna then both small and mixed frond habitat units in uncaged treatments (large + uncaged × small + uncaged, *p* < .0001; large + uncaged × mix + uncaged, *p* < .0001) (Figure 4, Table 5).

**TABLE 5:** Tukey's post hoc test for significant interaction between habitat unit morphology and predator access (cage treatment) on the abundance of epifauna (Table 1).

Contrast	Estimate	Z	*P*(<Z)
Mix + Full cage × Small + Full cage	−.14	−4.47	.0001*
Large + Full cage × Small + Full cage	.01	.27	.99
Large + Full cage × Mix + Full cage	.15	4.31	.0002
Mix + Uncaged × Small + Uncaged	−.18	−5.45	<.0001*
Large + Uncaged × Small + Uncaged	−.55	−15.37	<.0001*
Large + Uncaged × Mix + Uncaged	−.3	−9.29	<.0001*
Small + Full cage * Small + Uncaged	−.07236	−2.273	.2051
Mix + Full cage × Mix + Uncaged	.03627	1.100	.8815
Large + Full cage × Large + Uncaged	.49154	12.430	<.0001*
Large + Full cage × Mix + Uncaged	.18978	5.209	<.0001*
Large + Full cage × Small + Uncaged	−.06274	−1.777	.4809
Large + Uncaged × Mix + Full cage	−.33804	−9.293	<.0001*
Large + Uncaged × Small + Full cage	−.48193	−13.214	<.0001*
Mix + Full cage × Small + Uncaged	−.21625	−7.738	<.0001*

*Note*: Open‐cage treatments could not be included due to the loss of several large frond habitat units to suspected herbivory. ‘Small’, small frond double branch habitat unit; ‘Large’, large frond double branch habitat unit; ‘Mix’, mixed frond double branch habitat unit; ‘Full cage’, no predator access; ‘uncaged’, full predator access. Significant differences are indicated with *.

**FIGURE 5:**
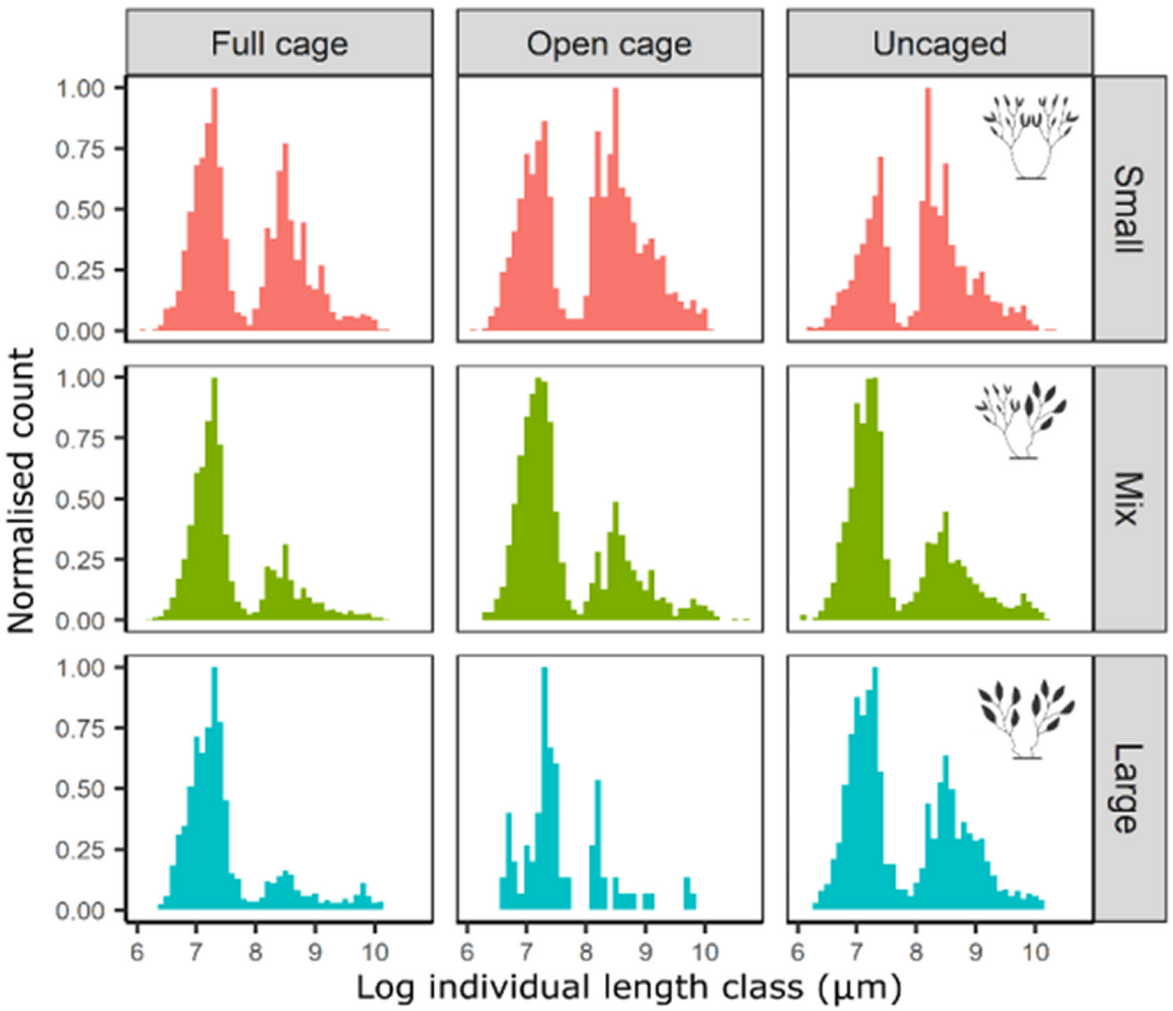
Size structure of epifaunal communities pooled from each algal morphology × caging treatment. Suspected herbivory meant that community size structure for open‐cage, large frond habitat unit (bottom centre panel) consists of only one treatment (see main text for more details). Different colours reflect frond size diversity of habitat units (small, large and mixed mean frond size). Y‐axis shows the number of individuals in each size bin normalised by the total number of individuals in the community. Epifauna were placed into size bins based on log‐transformed body length (μm).

**FIGURE 6:**
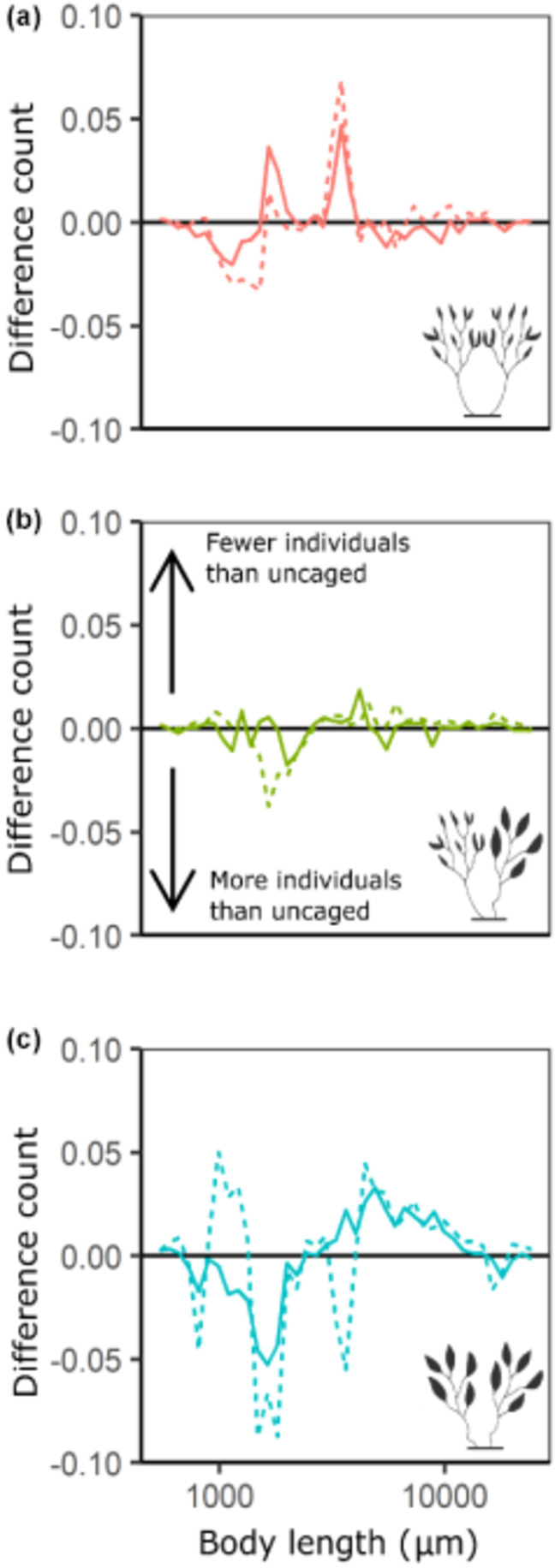
Delta plots showing size structure differences of epifaunal communities from uncaged (baseline, black horizontal line), open‐cage (dashed line) and full‐cage treatments (solid line). Negative values represent size bins that contained more individuals, while positive values represent bins that contained less individuals than uncaged treatments. Zero values represent no difference between cage treatments. Plots show size structure of epifaunal communities pooled for (a) small frond, (b) mixed frond and (c) large frond habitat units. Suspected herbivory means that community size structure for open‐cage, large frond habitat unit (c, dotted line) consists of only one treatment (see main text for more details). Epifauna were placed into size bins based on log‐transformed body length (μm).

There was no significant effect of predation on the mean body length or variance in body length of epifaunal communities (Table 6). Epifauna body size distributions were, however, strongly bimodal and comparisons of frequency histograms show that while there was no effect of habitat unit morphology or caging treatment on mean body size and variance in body size, there was variability in the size structure of epifaunal communities from different caging treatments and from habitat units of different morphologies (Figure 5). Delta plots showed predation risk had little influence on the size structure of communities from mixed frond habitat units (Figure 6b). In comparison, communities from large frond habitat units displayed highly variable size structures, with communities protected from predators (i.e. caged treatments) containing many more small individuals and many fewer larger individuals than were present in communities exposed to predators (i.e. uncaged treatments) (Figure 6c). The size structure of communities from small frond habitat units also displayed some variability in response to predators (Figure 6a).

**TABLE 6:** Results of models testing for the effect of habitat unit morphology and predator access (cage treatment) on mean body length and variance in body length of epifauna communities.

	Mean body length			Variance in body length		
	Df	Z	*P*(<Z)	Df	Z	*P*(<Z)
Cage treatment (C)	1,30	1.84	.25	1,30	.72	.22
Habitat unit morphology (HM)	2,30	.17	.94	2.30	.61	.46
C × HM	2,27	.74	.79	2,27	.55	.5

*Note*: Open‐cage treatments could not be included due to the loss of several large frond habitat units to suspected herbivory. This analysis therefore compares open cage treatments to uncaged treatments only. Mean body length was tested using an LM and variance in body length was tested using a GLM with a Gamma log link error distribution.

## 4 DISCUSSION

Previous studies demonstrate that habitat structure and predation interact to influence associated community structure. However, how these factors are meditated by morphological traits of the habitat are poorly known. We used naturally occurring morphological variability in the alga *S. vestitum* to create habitat units of specific morphotypes and found that intraspecific variation in frond size, a key morphological trait, strongly affected the epifaunal communities associated with macroalgae, irrespective of the size of habitat units. Contrary to our hypothesis, however, frond size traits did not interact, meaning that higher morphological diversity in the habitat (i.e. small and large fronds present within a single habitat unit) did not lead to higher abundances of colonising epifauna. Experimentally excluding predators showed that these patterns were likely the result of predation, with little effect of variation in habitat structure on epifauna in the absence of predation. In comparison, epifaunal communities varied strongly with habitat structure when subject to predation.

Comparisons of epifaunal community body size distributions supported these findings, with little variation found among caging treatments for both small and mixed frond habitat units. Predator access to communities on large frond habitat units, however, resulted in epifaunal communities with a lower proportion of small and more large individuals than those that were protected from predators. These results support our hypothesis that morphology affects the refuge value of macroalgae, suggesting that the structure of epifaunal communities associated with *S. vestitum* is the result of size‐selective predation of communities from macroalgae with large fronds, rather than epifauna preferentially selecting for macroalgae with small fronds. These findings highlight the significant impact habitat‐former traits can have on associated communities, but that these impacts occur only in the presence of predators and also vary with prey body size.

### 4.1 Influence of habitat traits on habitat‐former associated communities

In terrestrial systems, there is growing evidence that individual traits do not act in isolation. For example, floral traits such as colour, orientation and nectar quality are known to synergistically interact to affect pollinators (Gegear et al., 2017). In this study, however, we found no interaction between frond size and thallus size (i.e. habitat unit size/algal biomass), suggesting that the addition of extra biomass to our habitat units did not affect their value to associated communities. This was surprising, as double branch habitat units theoretically have more available habitat than single branch habitat units, with the additional space that occurs between algal branches. While several studies have found epifauna respond positively to interstitial spaces within algal habitats (Hacker & Steneck, 1990; Warfe et al., 2008), their usefulness to associated communities will likely be dependent on the presence and/or size of predators, as larger spaces may not exclude predators (Bartholomew et al., 2000; Warfe & Barmuta, 2004).

The abundance of epifauna colonising our habitat units was strongly related to *S. vestitum* frond size, highlighting the significant influence intraspecific variation in the traits of habitat‐formers has on associated communities. On land, intraspecific variation within host plants drives variability in resource quality and availability and will often lead to more diverse consumer communities (e.g. Crutsinger et al., 2006; Tielens & Gruner, 2020). Plant intraspecific variation can also affect community abundance, as in seagrass, where increased genetic diversity had a positive effect on the abundance of associated invertebrate communities following a disturbance event (Hughes & Stachowicz, 2004). Any variation in the communities associated with habitat formers will have significant implications for food web dynamics as these communities are an important trophic link between primary producers and higher trophic levels. Thus, intraspecific variability in the traits of habitat formers has the potential to have far reaching impacts, not just for habitat formers themselves, or the organisms that directly interact with them, but for entire communities.

Our mixed frond habitat units were found to host higher epifauna abundances than large frond habitat units, but less than small frond habitat units. This was surprising as the mixed frond habitat units were created to mimic naturally occurring intra‐individual variability often displayed by *S. vestitum* (Stelling‐Wood et al., 2020, 2021). We hypothesised that higher morphological variability within a habitat unit, representing more complex habitat, would positively correlate with the abundance of associated organisms (Chemello & Milazzo, 2002; Warfe et al., 2008) and would support more size‐diverse associated communities. This, however, was not the case in our study with our findings suggesting that increased heterogeneity in a habitat does not necessarily result in higher abundances of associated fauna. Similarly, habitat unit morphology also had no effect on the mean body length or variance in body length of associated communities. This again was surprising, as body size–habitat structure relationships have been found in multiple taxa and across multiple habitats types (e.g. amphipods in marine algae, Hacker & Steneck, 1990; spiders in terrestrial forests, Gunnarsson, 1992; fish in multiple marine landscapes, Nash et al., 2013). None of these studies, however, included the small body sizes we included in this study. We used a sieve size of 200 μm to ensure we captured harpacticoid copepods that are known to numerically dominate the communities associated with *Sargassum* (in addition to amphipods; Poore et al., 2000; Roberts & Poore, 2006). While many species of amphipod are known to associate preferentially with microhabitats that closely match their body size (Hacker & Steneck, 1990; Parker et al., 2001; Viejo, 1999), the small body size of copepods may reduce their interaction with the habitat meaning that they are unaffected by any variation in habitat structure. Our findings do, however, support the results of previous research which found frond size was negatively correlated with the abundance of epifauna across six species of algae (Stelling‐Wood et al., 2020).

### 4.2 Relative influence of abiotic habitat provisioning and predation risk on habitat former associated communities

Macrophytes on temperate reefs are important foraging grounds for invertivorous fish, but studies testing for the effects of fish predators on the abundance of epifauna have found varying results (Edgar & Aoki, 1993; Machado et al., 2019; Ndhlovu et al., 2021). Our results, demonstrating an interaction between morphological traits and predation risk, go some way to resolving these contradictory results. In the absence of predation, all habitat units appeared to be of largely equal value. However, when predators were present, large frond habitat units proved to be of low refuge value, consistently hosting lower abundances of epifauna. This confirms prior work demonstrating that the influence of habitat structure can be context dependent (Kichenin et al., 2013; Klecka & Boukal, 2014; Wardle et al., 2008), and demonstrates that predation can drive these variable effects.

The finding of low epifauna abundances on habitat units with large fronds was consistent across all our analyses, suggesting that high predation on macroalgae with large fronds drives epifauna abundance patterns on temperate reefs. Similar patterns have been documented in seagrasses, where Leber (1985) found only small differences in the abundance of amphipods between vegetated and unvegetated patches when predators were excluded, whereas in the presence of predators, strong differences were found between patches of moderate and high structural complexity. These results emphasise the importance of habitat formers beyond the provision of habitat, showing that the structure they provide can also significantly impact associated communities by moderating biotic interactions. Interestingly, however, our findings show that intraspecific trait variation can influence how effective habitat formers are at moderating these interactions.

While large frond habitat units provided low‐quality refuge to epifauna overall, the refuge value of this morphotype was also related to epifauna body size. Predator access altered the body size distributions of communities from large frond habitat units, leading to communities with a lower proportion of small‐bodied individuals and more large‐bodied individuals compared to communities excluded from predators. Similar patterns were not seen in epifaunal communities from habitat units of other morphotypes, suggesting that predation risk varied with body size only on large frond alga. This pattern may be the result of predators actively targeting small‐bodied individuals or could reflect a reduction in the ability of small‐bodied individuals to evade predators amongst the large fronds. Our findings are consistent with a similar study which found predation reduced the densities of epifauna on *Sargassum* disproportionately across different body sizes; however, this study instead found larger bodied individuals experienced high rates of predation (Edgar & Aoki, 1993).

In our study, all body sizes appear to have experienced roughly equal susceptibility to predation when on small and mixed frond habitat units, while on large frond morphotypes predation risk was instead disproportionately higher for small‐bodied individuals. McAbendroth et al. (2005) found that spatial divisioning in macrophytes was positively associated with a greater portion of small‐bodied individuals in associated communities. The authors suggested that smaller bodied organisms were favoured where macrophytes had smaller ‘inter‐vegetation gaps’, as larger bodied organisms likely found it more difficult to move around. In the presence of predators, however, this relationship becomes more complex as the value of habitat to prey will also be a function of predator size (Bartholomew et al., 2000). While we did not measure interstitial spaces in our experiment, we did observe variability in the density with which fronds occurred on the different morphotypes, with small frond morphotypes generally having densely packed fronds, while fronds often grew more sparsely on large frond morphotypes (Figure A1). Thus, it appears that the structure of our large frond morphotype was less effective than small and mixed frond morphotypes at excluding predators. This suggests that the susceptibility of prey individuals to predation is a function of both habitat structure and body size, with our results showing that on temperate reefs size‐selective predation can be mediated by variation in the frond size of habitat‐forming macroalgae.

By harnessing the natural morphological variability that occurs among individuals of *S. vestitum*, we demonstrated that habitat structure interacts with predation risk to structure associated epifaunal communities. We found that intraspecific variation in the frond size of algae only influenced epifaunal communities in the presence of predators, suggesting that the importance of macroalgae on temperate reefs lies in their capacity to mediate predator–prey interactions and is not the result of habitat provisioning. Furthermore, by examining body size, in addition to the abundance of associated organisms, we were able to show that habitat units of *S. vestitum* with large fronds provided low‐quality refuge from predation, but that the refuge value of these habitats was related to prey body size. Overall, these results demonstrate that the consideration of physical traits that describe habitat structure cannot be examined without the inclusion of other biotic factors (such as predation) if we are to fully understand the mechanisms that structure the communities associated with habitat‐forming organisms.

## AUTHOR CONTRIBUTIONS

**Talia P. Stelling‐Wood:** Conceptualization (equal); formal analysis (lead); investigation (lead); methodology (lead); visualization (lead); writing – original draft (lead); writing – review and editing (equal). **Alistair G. B. Poore:** Conceptualization (equal); methodology (equal); supervision (equal); writing – review and editing (equal). **A. Randall Hughes:** Conceptualization (supporting); writing – review and editing (equal). **Jason D. Everett:** Data curation (supporting); formal analysis (supporting); writing – review and editing (equal). **Paul E. Gribben:** Conceptualization (equal); methodology (equal); supervision (equal); writing – review and editing (equal).

## CONFLICT OF INTEREST STATEMENT

The authors declare no conflicts of interest.

## ETHICS STATEMENT

Collections were made with permission from permit P13/0007 issued by the Department of Primary Industries, New South Wales.

## Figures and Tables

**FIGURE A2:**
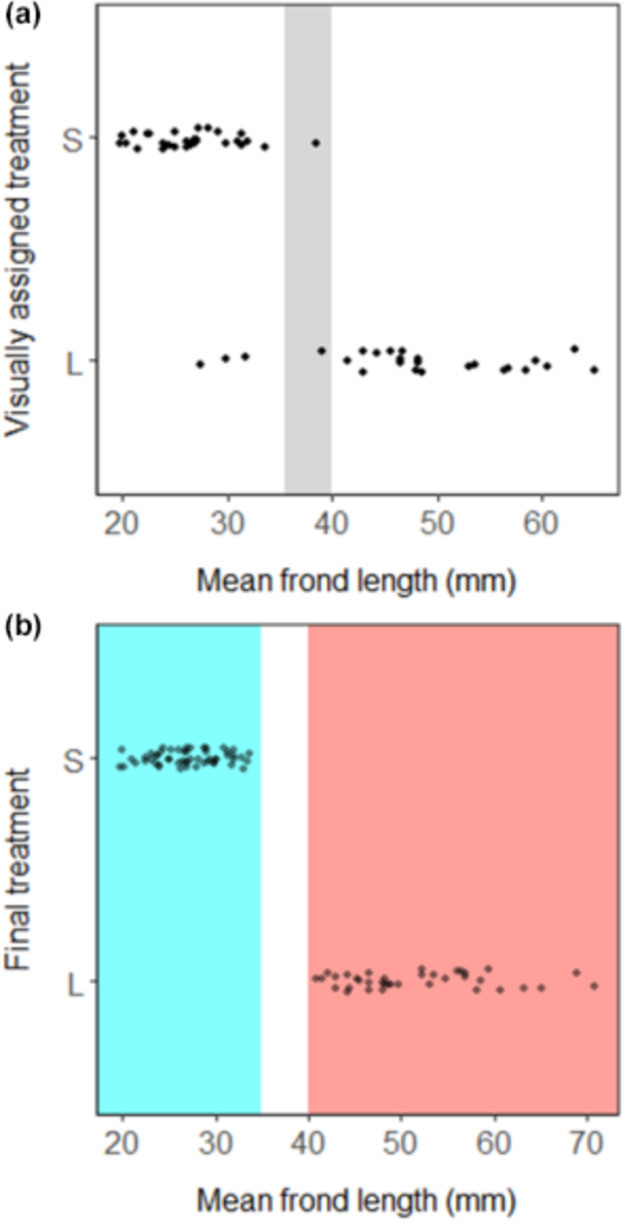
Plots shows the mean frond length of each algal branch used in all experiments, ‘S’ is branches that are assigned to small frond size treatments and ‘L’ is branches that are assigned to large frond size treatments. (a) Shows how algal branches were visually assigned into the different frond size treatments in the field. The grey‐shaded area is where the mean frond size of the two frond size treatments overlapped. To ensure frond size treatments were significantly different from each other any algal branches that fell within this region were removed from the experiment. (b) Shows final frond size treatment allocation used in the analyses of both experiments. Here, algal branches within the region of overlap have been removed, and those branches that were incorrectly assigned in the field were also reassigned into their correct morphotypes (i.e. algal branches assigned to the ‘L’ treatment but that fell to the left of the grey region were reassigned to ‘S’ treatment).

## Data Availability

Data are available on Dryad: https://doi.org/10.5061/dryad.g1jwstqvj.

## APPENDIX A

### A.1 Frond size treatment allocation

The visual allocation of all algal branches into frond size treatments (small frond morphotype and large frond morphotype, Figure A1) that were used in the construction of habitat units in the field were validated back in the laboratory after epifaunal communities had been removed. Ten fronds were randomly removed from each branch of algae and scanned using a Canon LIDE220 A4 flatbed scanner. The length of each frond was then measured from the scanned image using the image analysis software (ImageJ 1.50i; National Institute of Health). The average of all 10 fronds was used to establish mean frond size for each branch. These values were then plotted for small and large frond allocated branches (i.e. the mean frond size for branches allocated to small frond size treatments in the field was plotted on the same axis as the mean frond size for large treatments) (mixed frond size habitat units were not included in this plot). There was an area of overlap between the two frond size treatments where branches could have belonged to either frond size treatment that is, they had similar mean frond sizes (grey area, Figure A2). ‘Small’ frond branches were then therefore to be any branch with a mean frond length smaller than this area of overlap (i.e. less than or equal to 35 mm: blue‐shaded area, Figure A2). Similarly, ‘large’ frond treatments were taken to be algal branches with a mean frond length greater than the area of overlap (i.e. greater than or equal to 40 mm: pink‐shaded area, Figure A2). All branches with a mean frond size greater than 35 mm but less than 40 mm were excluded from further analyses. This meant also excluding the companion algal branch from further analyses if the excluded branch came from a double branch habitat unit. In total six branches fell within the overlap region, and therefore, all six habitat units were excluded from further analyses. Branches that fell outside the area of overlap (grey area) but on the wrong side of the plot (i.e. ‘large’ frond branches than fell to the left of the grey area, Figure A2) were reassigned to the correct frond size treatment (i.e. were reassigned as ‘small’ frond branches).

A further seven habitat units were lost to suspected herbivory and one cage was lost during the experiment (in total 14 habitat units were lost or excluded). This left a total of 50 habitat units remaining for analyses across both experiments. For the first experiment, 25 out of the original 28 habitat units were analysed, and for the second experiment, 41 out of the original 54 habitat units were analysed. Note, double branch habitat units from uncaged treatments were included in the analyses for both experiments.

## References

[ece310771-bib-0001] Bartholomew, A. , Diaz, R. J. , & Cicchetti, G. (2000). New dimensionless indices of structural habitat complexity: Predicted and actual effects on a predator^1^s foraging success. Marine Ecology Progress Series, 206, 45–58.

[ece310771-bib-0002] Bertness, M. D. , Gaines, S. D. , & Hay, M. E. (2001). Marine community ecology. Sinauer Associates.

[ece310771-bib-0003] Bruno, J. F. , Boyer, K. E. , Duffy, J. E. , Lee, S. C. , & Kertesz, J. S. (2005). Effects of macroalgal species identity and richness on primary production in benthic marine communities. Ecology Letters, 8(11), 1165–1174. 21352440 10.1111/j.1461-0248.2005.00823.x

[ece310771-bib-0004] Chemello, R. , & Milazzo, M. (2002). Effect of algal architecture on associated fauna: Some evidence from phytal molluscs. Marine Biology, 140(5), 981–990.

[ece310771-bib-0005] Coleman, M. A. , & Wernberg, T. (2017). Forgotten underwater forests: The key role of fucoids on Australian temperate reefs. Ecology and Evolution, 7(20), 8406–8418. 29075458 10.1002/ece3.3279 PMC5648665

[ece310771-bib-0006] Crutsinger, G. M. , Collins, M. D. , Fordyce, J. A. , Gompert, Z. , Nice, C. C. , & Sanders, N. J. (2006). Plant genotypic diversity predicts community structure and governs an ecosystem process. Science, 313(5789), 966–968. 16917062 10.1126/science.1128326

[ece310771-bib-0055] Denno, R. F. , Finke, D. L. , & Langellotto, G. A. (2005). Direct and indirect effects of vegetation structure and habitat complexity on predator‐prey and predator‐predator interactions. In P. Barbosa , & I. Castellanos (Eds.), Ecology of Predator‐Prey Interactions (pp. 211–239). Oxford University Press.

[ece310771-bib-0007] Edgar, G. J. , & Aoki, M. (1993). Resource limitation and fish predation: Their importance to mobile epifauna associated with Japanese Sargassum. Oecologia, 95(1), 122–133. 28313320 10.1007/BF00649515

[ece310771-bib-0008] Gee, J. , & Warwick, R. (1994). Metazoan community structure in relation to the fractal dimensions of marine macroalgae. Marine Ecology Progress Series, 103, 141–150.

[ece310771-bib-0009] Gegear, R. J. , Burns, R. , & Swoboda‐Bhattarai, K. A. (2017). “Hummingbird” floral traits interact synergistically to discourage visitation by bumble bee foragers. Ecology, 98(2), 489–499. 27864943 10.1002/ecy.1661

[ece310771-bib-0010] Gorsky, G. , Ohman, M. D. , Picheral, M. , Gasparini, S. , Stemmann, L. , Romagnan, J. B. , Cawood, A. , Pesant, S. , Garcia‐Comas, C. , & Prejger, F. (2010). Digital zooplankton image analysis using the ZooScan integrated system. Journal of Plankton Research, 32(3), 285–303.

[ece310771-bib-0011] Gribben, P. E. , Kimbro, D. L. , Vergés, A. , Gouhier, T. C. , Burrell, S. , Garthwin, R. G. , Cagigas, M. L. , Tordoff, Y. , & Poore, A. G. B. (2017). Positive and negative interactions control a facilitation cascade. Ecosphere, 8(12), e02065.

[ece310771-bib-0012] Gunnarsson, B. (1992). Fractal dimension of plants and body size distribution in spiders. Functional Ecology, 6, 636–641.

[ece310771-bib-0013] Hacker, S. D. , & Steneck, R. S. (1990). Habitat architecture and the abundance and body‐size‐dependent habitat selection of a phytal amphipod. Ecology, 71(6), 2269–2285.

[ece310771-bib-0014] Heather, F. J. , Stuart‐Smith, R. D. , Blanchard, J. L. , Fraser, K. M. , & Edgar, G. J. (2021). Reef communities show predictable undulations in linear abundance size spectra from copepods to sharks. Ecology Letters, 24, 2146–2154. 34291561 10.1111/ele.13844

[ece310771-bib-0015] Holling, C. S. (1992). Cross‐scale morphology, geometry, and dynamics of ecosystems. Ecological Monographs, 62(4), 447–502.

[ece310771-bib-0016] Hughes, A. R. , & Stachowicz, J. J. (2004). Genetic diversity enhances the resistance of a seagrass ecosystem to disturbance. Proceedings of the National Academy of Sciences, 101(24), 8998–9002. 10.1073/pnas.0402642101 PMC428461 15184681

[ece310771-bib-0017] Jones, C. , Lawton, J. , & Shachak, M. (1994). Organisms as ecosystem engineers. Oikos, 69(3), 373–386.

[ece310771-bib-0018] Khanaposhtani, M. G. , Kaboli, M. , Karami, M. , & Etemad, V. (2012). Effect of habitat complexity on richness, abundance and distributional pattern of forest birds. Environmental Management, 50(2), 296–303. 22661015 10.1007/s00267-012-9877-7

[ece310771-bib-0019] Kichenin, E. , Wardle, D. A. , Peltzer, D. A. , Morse, C. W. , & Freschet, G. T. (2013). Contrasting effects of plant inter‐and intraspecific variation on community‐level trait measures along an environmental gradient. Functional Ecology, 27(5), 1254–1261.

[ece310771-bib-0020] Klecka, J. , & Boukal, D. S. (2014). The effect of habitat structure on prey mortality depends on predator and prey microhabitat use. Oecologia, 176(1), 183–191. 25085443 10.1007/s00442-014-3007-6

[ece310771-bib-0021] Lapointe, L. , & Bourget, E. (1999). Influence of substratum heterogeneity scales and complexity on a temperate epibenthic marine community. Marine Ecology Progress Series, 189, 159–170.

[ece310771-bib-0022] Laughlin, D. C. (2014). The intrinsic dimensionality of plant traits and its relevance to community assembly. Journal of Ecology, 102(1), 186–193.

[ece310771-bib-0023] Leber, K. M. (1985). The influence of predatory decapods, refuge, and microhabitat selection on seagrass communities. Ecology, 66(6), 1951–1964.

[ece310771-bib-0024] Lenth, R. , Singmann, H. , Love, J. , Buerkner, P. , & Herve, M. (2018). Emmeans: Estimated marginal means, aka least‐squares means. R package version, 1(1), 3.

[ece310771-bib-0025] Lloyd, H. B. , Cruz‐Motta, J. J. , Glasby, T. M. , Hutchings, P. A. , & Gribben, P. E. (2020). Unusual but consistent latitudinal patterns in macroalgal habitats and their invertebrate communities across two countries. Diversity and Distributions, 26(8), 912–927.

[ece310771-bib-0026] MacArthur, R. H. , & MacArthur, J. W. (1961). On bird species diversity. Ecology, 42(3), 594–598.

[ece310771-bib-0027] Machado, G. B. , Ferreira, A. P. , Bueno, M. , Siqueira, S. G. , & Leite, F. P. (2019). Effects of macroalgal host identity and predation on an amphipod assemblage from a subtropical rocky shore. Hydrobiologia, 836(1), 65–81.

[ece310771-bib-0028] McAbendroth, L. , Ramsay, P. , Foggo, A. , Rundle, S. , & Bilton, D. (2005). Does macrophyte fractal complexity drive invertebrate diversity, biomass and body size distributions? Oikos, 111(2), 279–290.

[ece310771-bib-0029] Nash, K. L. , Graham, N. A. , Wilson, S. K. , & Bellwood, D. R. (2013). Cross‐scale habitat structure drives fish body size distributions on coral reefs. Ecosystems, 16(3), 478–490.

[ece310771-bib-0030] Ndhlovu, A. , Lathlean, J. A. , McQuaid, C. D. , & Seuront, L. (2021). Indirect effects shape macroalgal epifaunal communities. Ecology and Evolution, 11, 15141–15152. 34765166 10.1002/ece3.8195 PMC8571615

[ece310771-bib-0031] Parker, J. D. , Duffy, J. E. , & Orth, R. J. (2001). Plant species diversity and composition: Experimental effects on marine epifaunal assemblages. Marine Ecology Progress Series, 224, 55–67.

[ece310771-bib-0032] Phillips, P. C. , & Arnold, S. J. (1989). Visualizing multivariate selection. Evolution, 43(6), 1209–1222. 28564514 10.1111/j.1558-5646.1989.tb02569.x

[ece310771-bib-0033] Pistón, N. , de Bello, F. , Dias, A. T. , Götzenberger, L. , Rosado, B. H. , de Mattos, E. A. , Salguero‐Gómez, R. , & Carmona, C. P. (2019). Multidimensional ecological analyses demonstrate how interactions between functional traits shape fitness and life history strategies. Journal of Ecology, 107(5), 2317–2328.

[ece310771-bib-0034] Poore, A. G. (2005). Scales of dispersal among hosts in a herbivorous marine amphipod. Austral Ecology, 30(2), 219–228.

[ece310771-bib-0054] Poore, A. , Watson, M. , de Nys, R. , Lowry, J. , & Steinberg, P. (2000). Patterns of host use among alga‐ and sponge‐associated amphipods. Marine Ecology Progress Series, 208, 183–196. 10.3354/meps208183

[ece310771-bib-0035] Raffaelli, D. , Hall, S. , Emes, C. , & Manly, B. (2000). Constraints on body size distributions: An experimental approach using a small‐scale system. Oecologia, 122(3), 389–398. 28308290 10.1007/s004420050045

[ece310771-bib-0036] Roberts, D. A. , & Poore, A. G. B. (2006). Habitat configuration affects colonisation of epifauna in a marine algal bed. Biological Conservation, 127(1), 18–26.

[ece310771-bib-0037] Robson, B. , Barmuta, L. , & Fairweather, P. G. (2005). Methodological and conceptual issues in the search for a relationship between animal body‐size distributions and benthic habitat architecture. Marine and Freshwater Research, 56(1), 1–11.

[ece310771-bib-0038] Romero, G. Q. , Gonçalves‐Souza, T. , Vieira, C. , & Koricheva, J. (2015). Ecosystem engineering effects on species diversity across ecosystems: A meta‐analysis. Biological Reviews, 90(3), 877–890. 25174581 10.1111/brv.12138

[ece310771-bib-0039] Schwinghamer, P. (1981). Characteristic size distributions of integral benthic communities. Canadian Journal of Fisheries and Aquatic Sciences, 38(10), 1255–1263.

[ece310771-bib-0040] Stelling‐Wood, T. P. , Gribben, P. E. , & Poore, A. G. B. (2020). Habitat variability in an underwater forest: Using a trait‐based approach to predict associated communities. Functional Ecology, 34(4), 888–898.

[ece310771-bib-0041] Stelling‐Wood, T. P. , Poore, A. G. B. , & Gribben, P. E. (2021). Shifts in biomass and structure of habitat‐formers across a latitudinal gradient. Ecology and Evolution, 11, 1–12. 10.1002/ece3.7714 PMC8258212 34257931

[ece310771-bib-0042] Teagle, H. , Hawkins, S. J. , Moore, P. J. , & Smale, D. A. (2017). The role of kelp species as biogenic habitat formers in coastal marine ecosystems. Journal of Experimental Marine Biology and Ecology, 492, 81–98.

[ece310771-bib-0043] Thomsen, M. S. , Altieri, A. H. , Angelini, C. , Bishop, M. J. , Bulleri, F. , Farhan, R. , Frühling, V. M. , Gribben, P. E. , Harrison, S. B. , He, Q. , & Klinghardt, M. (2022). Heterogeneity within and among co‐occurring foundation species increases biodiversity. Nature Communications, 13(1), 1–9. 10.1038/s41467-022-28194-y PMC8803935 35102155

[ece310771-bib-0044] Tielens, E. K. , & Gruner, D. S. (2020). Intraspecific variation in host plant traits mediates taxonomic and functional composition of local insect herbivore communities. Ecological Entomology, 45(6), 1382–1395.

[ece310771-bib-0045] Toscano, B. J. , & Griffen, B. D. (2013). Predator size interacts with habitat structure to determine the allometric scaling of the functional response. Oikos, 122(3), 454–462.

[ece310771-bib-0046] Viejo, R. M. (1999). Mobile epifauna inhabiting the invasive Sargassum muticum and two local seaweeds in northern Spain. Aquatic Botany, 64(2), 131–149.

[ece310771-bib-0047] Wang, A. Y. , Epperson, W. , DeLine, R. A. , & Drucker, S. M. (2022). Diff in the loop: Supporting data comparison in exploratory data analysis. In *CHI Conference on Human Factors in Computing Systems*.

[ece310771-bib-0048] Wardle, D. A. , Lagerström, A. , & Nilsson, M. C. (2008). Context dependent effects of plant species and functional group loss on vegetation invasibility across an Island area gradient. Journal of Ecology, 96, 1174–1186.

[ece310771-bib-0049] Warfe, D. , Barmuta, L. , & Wotherspoon, S. (2008). Quantifying habitat structure: Surface convolution and living space for species in complex environments. Oikos, 117(12), 1764–1773.

[ece310771-bib-0050] Warfe, D. M. , & Barmuta, L. A. (2004). Habitat structural complexity mediates the foraging success of multiple predator species. Oecologia, 141(1), 171–178. 15300485 10.1007/s00442-004-1644-x

[ece310771-bib-0051] Wright, J. T. , & Gribben, P. E. (2017). Disturbance‐mediated facilitation by an intertidal ecosystem engineer. Ecology, 98(9), 2425–2436. 28628212 10.1002/ecy.1932

[ece310771-bib-0052] Zamzow, J. P. , Amsler, C. D. , McClintock, J. B. , & Baker, B. J. (2010). Habitat choice and predator avoidance by Antarctic amphipods: The roles of algal chemistry and morphology. Marine Ecology Progress Series, 400, 155–163.

[ece310771-bib-0053] Zuur, A. F. , Ieno, E. N. , Walker, N. J. , Saveliev, A. A. , & Smith, G. M. (2009). GLM and GAM for count data. In Mixed effects models and extensions in ecology with R (pp. 209–243). Springer.

